# Innovative Activity of the Service Sector of the EU Member States

**DOI:** 10.1007/s13132-023-01143-w

**Published:** 2023-05-11

**Authors:** Kamil Decyk

**Affiliations:** Faculty of Economic Sciences, University of Warmia and Mazury, Olsztyn, Poland

**Keywords:** Expenditure on innovative activities, R&D, Effects of innovative activities, Service sector, EU countries, Effectiveness of innovative activities, O30, O32, O52

## Abstract

Expenditures on innovative activities, including R&D, play a key role in introducing innovations and consequently in the level of innovativeness. The aim of the research was to assess the level of innovative activity in the service sector in the Member States of the European Union (EU), in terms of incurred expenditure and achieved effects. To achieve the given aim various methods were used, including: monographic method, analysis and criticism of the literature, index and statistical analyses. The implementation of the assumed goal was carried out on the basis of two groups of indicators — effects and expenditure incurred on innovative activities. The research procedure was carried out in three stages: detailed assessment of the indicators, identification of similarities within the studied countries and determination of correlations occurring in them, and assessment of the synthetic Perkal’s indicator regarding outlays and effects. The results of the research provided information on the level of the use of inputs in relation to the effects in the service sector of individual EU countries. Countries ranked high in the innovativeness ranking (Denmark, France) are characterized by higher outlays and the effects achieved as a result of them, as well as show favorable or neutral relations between them. A high level of effects in relation to the expenditure was also observed in Malta, Spain, and Croatia. The results of the research constitute valuable information on the effectiveness of the use of expenditure on innovative activities, especially significant from the point of view of service entities from countries with a low level of innovative activity. These enterprises should draw on good practices and experience and even try to cooperate in the creation of innovations with companies from countries with a high level of innovative activity. Consequently, such practices will contribute to a more dynamic and sustainable development of innovativeness in the service sector of all EU.

## Introduction

The legitimacy of undertaking research work in the area of the issues discussed in the study is related to two current trends in the global economy. The first one concerns the importance (including economic) of the service sector, which is indicated, inter alia, by the World Bank (2021). ( According to his data, in 2018, the service sector accounted for 64.1% value added (% of gross domestic product — GDP). In addition, the literature on the subject (Cusumano et al., 2006) indicates that services provide opportunities to achieve long-term and more stable sources of income that allow enterprises to survive during a downturn.

The service sector and the economy function in a certain symbiosis. On the one hand, its impact on economic development requires a significant reduction in state intervention. On the other hand, thanks to the presence of consumer services, but primarily government services, service enterprises have practically limited to a minimum opportunities for economic development, as well as favorable conditions for development within activities involving innovative technologies (Riddl & Ott; Mark & Kravisem in: Cyrek, 2005). This is due, firstly, to the presence of government services, but not only to consumer services (which have limited possibilities of evolution), and, secondly, from favorable development conditions of enterprises undertaking activities in the field of innovative technologies.

The second reason and trend confirming the legitimacy of conducting research in the area of ​​the discussed issues is the growing and well-established role of innovativeness, which is widely recognized as one of the significant sources of competitiveness and even a key factor in stimulating economic growth. Service companies to become more competitive on the European market (Dobrzański et al., 2021; Hidalgo & D'Alvano, 2014; Li et al., 2018; Rua & Franca, 2017) should direct efforts towards the so-called “service innovations” related to social and business changes in companies (Gallouj et al., 2015), but not focus only on implementing innovations in their general understanding (Gonçalves et al., 2017; Jones & Basso, 2017). In the field of innovation-related activities, a special role in the development of competitiveness and increasing the attractiveness of the sector is assigned to expenditures on research and development (R&D), introduced innovations, but also factors independent of innovative activity, such as productivity (Satrovic et al., 2021). Among the above-mentioned determinants of competitiveness, the first two are particularly interesting in the context of the discussed issues. Expenditures on R&D activities, which together with the effects achieved as a result of their application (e.g., innovation), play a key role in economic growth and in the development of the knowledge-based economy (Odrobina, 2016; Ramadani et al., 2013). The literature on the subject also points to expenditures and innovation, as there is a complex, non-linear relationship between them, in which technological progress must be preceded by systematic activity in their scope (Ramadani et al., 2013).

Due to the above and from a cognitive and empirical point of view, an extremely interesting and important issue is the one that integrates aspects related to both the service sector and innovativeness. For this reason, the study searched for a solution to the research problem relating to the broadly understood role and importance of innovativeness in the service sector in the economies of the European Union (EU) countries in the twenty-first century.

The first chapter presents theoretical assumptions and reviews the literature on the discussed issues. The next one focuses on the methodological assumptions of the conducted research. The purpose and subject of the research were taken into account, and the research methods used were described in detail. The third chapter of the study is devoted to the discussion of the obtained results. Analyzes and interpretations were made on the basis of which summarizing conclusions were presented. Several important conclusions were identified, as well as the practical implications of the research and the possibility of its continuation in the future.

## Literature Review

The scope of innovative activities covers all kinds of development, financial and commercial activities that are undertaken by enterprises and which are aimed at creating innovation. This activity does not always have to be successful, because often its finalization for objective reasons may be postponed or even interrupted. For this reason, in practice, it is possible to speak, evaluate, and analyze innovative activity in relation not only to innovative economic entities, but all that function in a given economy (OECD, 2018). Such a comprehensive approach to both innovative and non-innovative entities is necessary, because the data on innovative activity has value in itself from the point of view of expenditures effectiveness, which can be related to innovations both directly and indirectly (OECD, 2018).

The inherent elements of innovative activity, on the basis of which it can be assessed, are, on the one hand, input components, which include, among others, outlays (expenses) on innovation, including R&D activity. On the other hand, there are exit elements that are extremely diverse, and their selection in many cases depends on the purpose and context of the entire research (Bilbao-Osorio & Rodriguez-Pose, 2004; Carlson, 2011; Firlej, 2019). The effects of innovative activity may include aspects related to the improvement of product quality, but also, apart from strictly product changes, streamlining work, and improving production efficiency (Babuchowska & Marks-Bielska, 2021). In terms of expenditures on innovative activities, two criteria are distinguished classification. The first of them is based on the so-called the accounting method for collecting data on innovation expenditures, according to which the expenditures on R&D (internal R&D costs) and other innovation activities are differentiated, including costs of own personnel, services purchased from other entities, investment expenditures (tangible fixed assets and intangible assets), or costs of materials. The second classification criterion distinguishes the types of innovative activities broken down by their specific types, important from the point of view of innovation. It is this classification that was adopted as the methodological basis for the research in this study. According to it, 7 categories of expenditures are distinguished, broken down by activity (OECD, 2018):

- Research and development
- Engineering, design and other creative activities
- Marketing and brand building
- Related to intellectual property
- Related to employee training
- Related to software development and databases
- Related to the acquisition or lease of property, plant, and equipment

Expenditures on R&D activities are often a separate subject of conducted analyzes. They are divided into government expenditures (Government Expenditures on R&D (GERD)) and incurred by the enterprise sector (Business Expenditures on R&D (BERD)). Governments of many countries are trying to implement an effective R&D investment policy to facilitate innovation and thus improve living standards and generate economic growth, which is good practice in terms of innovation development (Bilbao-Osorio & Rodriguez-Pose, 2004).

From the point of view of the service sector, a comprehensive approach to expenditures is more reasonable than the presented division of expenditures into GERD and BERD. This is due to the fact that service companies focus more attention on other types of innovative activities than on R&D. Additionally, as indicated in the literature (Geodecki, 2014), the assumption of expenditures on R&D and other measures of activity in this area, as a measure of enterprise innovativeness, is not entirely correct due to the fact that the research activity itself — development — is a way of solving the problem and not a factor of the company’s innovativeness.

The second inherent element influencing the level of innovative activity, apart from expenditures, is the effects, the measures of which may be, among others, the number of patent applications, industrial designs, trademarks, etc. The problem in the case of measurement based on patents is the fact that only some innovative works, knowledge, and technology are recorded in the patent (Voutsinas et al., 2018). Moreover, as noted by Nowacki and Staniewski (2012), as well as Gallouj and Windrum (2009), acquiring patents, as well as the degree of implementation of new technologies or the level of commercialization of R&D results, is much easier apply to the sphere of production rather than services. In the case of the service sector such as, IT, accounting and management, advertising and market research, and legal services, only trademarks that are used by innovative service enterprises more often than the aforementioned patents may be of particular importance for measuring the effects of innovative activities (Gotsch & Hipp, 2012). For this reason and also due to the specific nature of the services sphere, it seems that the most optimal solution is to use universal indicators for measuring innovativeness. These include, among other indices of innovative, non-innovative, and innovative activity enterprises, or the percentage of companies that have introduced product innovations or business processes (Carayannis & Grigoroudis, 2014). The last of these measures is of particular importance from the perspective of services. As indicated in the literature, the feature of services such as the inability to separate the time and place of production and consumption means that building relationships plays a key role between the service provider and the customer. This clearly shows that an innovative approach to services is important not only at the stage of designing or creating a service, but much more in contact with the recipient and in the ability to build relationships with them. In this case, business process innovations (marketing, organizational) are much more important than product innovations (Gallouj, 2002; Miles, 2011). Another indicator related to effects (measure, indicator), apart from the mentioned ones, may be the one related to revenues. In service enterprises, attention is paid to whether the new service proposed by the enterprise contributes to the improvement of its efficiency or the increase in sales revenues (Kozioł, 2009). In the literature on the subject, measures relating to revenues are often presented in the form of the level of revenues of new or modernized products in relation to their total value (Czubała, 2015; Tajer, 2015).

For a comprehensive assessment of innovative activity, it is important to analyze and diagnose not only expenses, but also to compare it with its effects. The degree of utilization of expenditures on innovation in conjunction with the analysis of the results is referred to as efficiency. A satisfactory level that allows for a positive assessment of innovative activity is a favorable relation of effects to incurred expenditures. Therefore, the goal is to constantly improve the relationship between these values. In this respect, innovative activities may be provided, such as creating and developing innovative potential, but also dynamizing the innovation process, which may also improve cooperation and contacts with customers (Kokot, 2016).

In the current literature review and empirical work, you can only find studies that identify and assess the relationship between innovation and expenditures, but only those dedicated to R&D. An example is the research on only R&D expenditures carried out by Sinoi (2021). They identified that in all seven tested models, expenditures on R&D (in various sectors of the economy) played an important role in supporting innovation. Positive and statistically significant coefficients were identified. The empirical material presented by Cruz-Cázares et al., (2013) also confirmed the positive relationship between R&D effectiveness and firm performance. The above-mentioned studies did not include, for example, works not strictly related to R&D. Outlays relating to other (except R&D) innovative activities (Bilbao-Osorio & Rodriguez-Pose, 2004; Pegkas et al., 2019) are extremely rare subject of studies. A significant research gap was observed in this respect. This gap was filled by the content, research, analyzes, and conclusions contained in this work.

## Methodology

### Basic Methodological Assumptions

The aim of the research was to assess the level of innovative activity in the service sector in the EU Member States in terms of incurred expenditure and achieved effects. In its scope, all expenditures on innovative activities (including R&D expenditures) were analyzed. To achieve the research goal, three auxiliary questions were used to support the research, which were also specific objectives of the research. The answers to the following questions were sought: What was the level of expenditures on innovative activities (also on R&D) in the EU Member States in 2018 and in the entire service sector? The second auxiliary question concerned the effects obtained as a result of the involvement of the aforementioned expenditures in countries and in the sector and was: What was the level of effects obtained as a result of expenditures incurred on innovative activities? The last detailed objective concerned the assessment of the level of use of inputs in relation to the obtained effects of innovative activity.

To achieve the assumed research goal, a number of research methods were used, which were necessary both at the stage of formulating basic methodological assumptions and during the development and analysis of the obtained empirical material. The basic method used to define the research area, on the basis of which the research goal was also formulated, was the method of analysis and critique of international literature. The monographic method was the second method supporting to a large extent the theoretical foundations of the work, but also allowing to define the methodological foundations for conducting research on broadly understood innovativeness. The Oslo Manual from 2018 was mainly used in its scope. The fourth and at the same time the latest edition is a continuation of the valuable, recognized and appreciated around the world series of Oslo Manuals, launched in 1992. The information contained in these manuals contains widely used and constantly updated methodology for researching innovativeness at the macro-, meso-, and microeconomic level. Both the latest 2018 edition and its previous versions provide a synthetic and unified approach to the principles of collecting and interpreting innovation data.

The third method helpful in achieving the set research goal was the indicator methods widely used in the research. Applied in the empirical part to a large extent, it allowed to assess the level of innovative activity of the service sector in terms of both inputs and the resultant effects.

### Data Collection and Sampling

The subject of research was the EU service sector. In line with the methodological approach presented by the Central Statistical Office in Poland (in Polish GUS), services include the following sections: G, H, J, K, and M. In the service sector, however, only some of the sections mentioned above are included. For example, from sector G, the statistics include only Sect. 46 (*Innovative activity of enterprises…,* 2020). A much broader approach to the service sector is presented in the Eurostat methodology. In the Structural Business Statistics database dedicated to service companies (sbs_na_serv), the H-N sectors are distinguished, as well as the S95 sectors, without distinguishing between individual departments. For the purposes of the study, the approach of the Central Statistical Office and Eurostat was compiled, and the availability of data in the European statistical database Eurostat was taken into account. As a result, the service sector included the entire G-N sectors, excluding section S95, for which data on innovative activity were not available. The service sector of all EU countries was analyzed. However, due to the lack of data on the expenditures of individual types of innovative activity, the number of countries was reduced to 10 in which the data was complete.

The subject of the research was the expenditures on innovative activities with a distinction between them into individual types of activity and the effects of innovative activities. The subject approach to the research problem allowed, inter alia, implementation of the specific objectives assumed in the research procedure.

### The Course of the Research Procedure

The research procedure aimed at achieving the assumed main goal was carried out in three stages, using the above-mentioned methods of data analysis:

1. Assessment of innovative activity in terms of expenditure on innovative activities and the effects obtained as a result of their use — ratio analysis
2. Assessment of the similarities and differences between the service sectors of the surveyed countries — cluster analysis using the Ward’s method, as well as the estimation of the Spearman’s rank correlation coefficient
3. Assessment of the degree of the obtained effects in relation to the inputs used, as well as a synthetic assessment of the level of innovative activity in this area — ratio analysis based on the Perkal’s indicator (PI) and the results of the cluster analysis

The first stage of the research was the evaluation of innovative activity. In its scope, a detailed analysis of indicators related to expenditures on innovative activities and the effects obtained as a result of their application were used. The group of indicators related to expenditures on innovative activities includes:

X_1_ – expenditures on innovation (in total) — including R&D activity — per service enterprise.

X_2_ – R&D expenses per one company.

X_3_ – expenditures on innovation (excluding those related to R&D) per one service company.

X_4_ – activities related to the acquisition or lease of property, plant and equipment, expressed in euro per service enterprise.

X_5_ – software development and database activities, expressed in euro per service company.

X_6_ – marketing activities and brand value building, expressed in euro per service enterprise.

X_7_ – activities related to the training of employees, expressed in euro per service enterprise.

X_8_ – activities related to the intellectual property, expressed in euro per service enterprise.

X_9_ – engineering, design and other creative activities, expressed in euro per service enterprise.

In the set of indicators describing the effects of the conducted innovative activity, seven measures were identified and assessed:

X_10_ – innovative enterprises as a percentage of all service companies.

X_11_ – enterprises active and innovative only in the scope of a product and/or process other than that related to the production of goods and services among entities from the entire sector.

X_12_ – enterprises that introduced business process innovations in relation to all service enterprises.

X_13_ – enterprises that have introduced product innovations in relation to enterprises from the entire service sector.

X_14_ – innovative enterprises as a percentage of all enterprises in the analyzed sector.

X_15_–X_17_ – revenues from the sale of new and modernized products (including services) in relation to total revenues, expressed in euro per service entity, taking into account the scale of new changes:

X_15_ – new or significantly improved for the market only.

X_16_ – new or significantly improved for the company only.

X_17_ – unchanged or slightly modified.

As a result of the first stage of research, the innovative activity was initially assessed; the EU countries were differentiated and grouped according to the level of individual, detailed indicators of innovative activity.

In the second stage of the research, the statistical method in the field of cluster analysis, combined with the Ward’s method, was used to achieve the goal, thanks to which it was possible to identify differences and similarities between the service sectors of the surveyed countries. The Euclidean distance was used as the measure of the distance between the studied countries. The results are presented in a graphical form (on a dendrogram) on the so-called binary tree, with a distinction between individual countries. This tree was used to aggregate the objects into clusters (sets, groups, clusters) according to the similarity between them (Chrobocińska, 2021; Wierzbicka, 2020). Based on the analysis of clusters and the dendrogram, it was possible to identify clusters of countries with similar outlays and the effects of innovative activities. At the same time, it should be added that the procedure for identifying the number of clusters was determined subjectively, based on the visual analysis of the dendrogram. When verifying the collections, the greatest effort was made to ensure that their number was not too large and that they were all distant from each other. In order to confirm the correctness of the identified clusters, the Spearman’s rank correlation coefficient was used as complementary and supportive measures. Correlations between countries that had previously been subjectively classified to individual clusters were calculated. The advantage of using the Spearman correlation index is that the data does not need to be normally distributed, and the computation is based on ranks, not values. The significance level of *p* = 0.95 was adopted for the interpretation of the correlation between countries in individual clusters. For the description and interpretation of the significant correlation, the following scale was adopted, determining the strength of the relationship between individual countries:

r_xy = 0 uncorrelated countries

(0–0.100) – very weak correlation.

 < 0.100–0.300) – weak correlation.

 < 0.300–0.500) – average correlation.

 < 0.500–0.700) – high correlation.

 < 0.700–0.900) – very high correlation.

 < 0.900–1.000) – almost full correlation.

r_xy = 1 – full correlation

The implementation of the second stage of the research made it possible to group the countries according to similarities and differences, first in terms of the expenditures incurred and secondly in terms of the effects achieved. The analysis of the binary tree allowed for the initial systematization and illustration of the general situation in the field of innovative activity. The use of the Spearman’s rank correlation coefficient allowed for an objective confirmation of the results of cluster analysis, which were burdened with some subjectivity, e.g., in terms of classification to clusters. Together, the first and second stages of the research made it possible to obtain answers to the first two auxiliary questions relating to the level of expenditures and the effects of innovative activity.

In the third stage of the research, on the basis of the presented detailed measures of innovative activity, Perkal’s indicator (PI) were calculated in terms of expenditures and outlays in individual countries. The use of this indicator allowed for a synthetic assessment of the level of innovative activity and also allowed for the assessment of the obtained effects in relation to the degree of expenditures in the service sector of the surveyed countries involved in innovative activities. The PI level was defined using a method based on the parameters of descriptive statistics such as mean and standard deviation (Table 1). The simultaneous use of the Perkal’s indicators and the method of grouping EU countries made it possible to determine the level of inputs and effects and thus contributed to the achievement of the assumed research goal (evaluation of the innovative activity of EU countries).

**Table 1:** Criteria for grouping countries according to the effects/inputs of innovative activities

Level	Grouping basis	Perkal’s index level
I	${\mathrm{d}}_{\mathrm{i}}\ge \overline{\mathrm{d} }+{\mathrm{S}}_{\mathrm{d}}$	high
II	$\overline{\mathrm{d} }<{\mathrm{d}}_{\mathrm{i}}\le \overline{\mathrm{d} }+{\mathrm{S}}_{\mathrm{d}}$	medium
III	$\overline{\mathrm{d} }-{\mathrm{S}}_{\mathrm{d}}<{\mathrm{d}}_{\mathrm{i}}\le \overline{\mathrm{d} }$	low
IV	${\mathrm{d}}_{\mathrm{i}}<\overline{\mathrm{d} }-{\mathrm{S}}_{\mathrm{d}}$	very low

Source: own elaboration on the basis of Wysocki & Lira (2005)

${\mathrm{d}}_{\mathrm{i}}$ — value of the service sector index of a given country

$\overline{\mathrm{d} }$ — the average value of the indicator in the entire study group

${\mathrm{S}}_{\mathrm{d}}$ — standard deviation of the index in the entire study group

The third stage of the research made it possible to assess the degree of use of inputs in relation to the results obtained as a result. It also made it possible to identify countries with a situation requiring improvement in the field of innovative activities, as well as those with a positive level.

It should be mentioned that conducting a simultaneous assessment of outlays and the results achieved as a result of them is quite complicated due to the fact that there is a time separation between them, reaching even several dozen years. The solution to this problem is provided by Sawulski (2018) in Adamczyk (2018), presenting two acceptable methods of data collection. The first one, used in this study, assumes the omission of time delays in the occurrence of effects. In the research, the data on both inputs and effects came from 2018. The second consists in comparing the results of innovative activity with the amount of expenditures from several years ago.

## Research Results

### Diagnosis of Innovative Activity in Terms of Incurred Expenses and Achieved Effects

As mentioned earlier, the assessment of the level of innovative activity in the service sector of the EU Member States was made mainly on the basis of indicators including inputs (X_1_–X_9_) and effects (X_10_–X_17_) obtained as a result of them. In the research procedure, an extensive analysis and interpretation of the results were carried out, in which the values of individual (detailed, partial) indicators were first observed, with a distinction between the EU countries.

When analyzing data on expenditures on innovative activities, the countries surveyed were systematized according to value of decreasing expenditures on innovation in total and the indicators are expressed in EUR per one enterprise (Table 2). Based on the presented data, it can be concluded that Denmark and France were the countries where the highest total expenditures on innovative activities (including R&D) per one company were recorded, respectively, 24871 EUR and 14954 EUR. It was respectively over three times more and more than 2.5 times more than in Malta, third in the classification. It was influenced by the level of expenditures on R&D, which in France was more than seven times higher (10,993 EUR/company) than in Malta (1 543 EUR/company). On the other hand, R&D expenditures by the Danish service sector exceeded the expenditures in Malta almost thirteen times. It should be mentioned here that from among the surveyed countries, only Denmark was included among the “innovation leaders” according to 2018 innovativeness ranking — 3rd place in the EU. France, on the other hand, was recognized as a “strong innovator” and classified in this ranking on the 11th place (Europejski Ranking Innowacyjności, 2018, 2018). The lowest expenditures on innovative activities (with R&D) were recorded in Portugal (1 376), including however a relatively large amount of expenditures on R&D activities alone, as much as 69.3% (953 EUR/company). At the same time, it should be noted that the lowest expenditures on R&D was incurred by service companies from Croatia (682 EUR/company).

**Table 2:** Innovation activity expenditures by activity in the EU service sector in 2018 (euro/company)

No	Country	Expenditures on innovation (with R&D)	Expenditures on R&D activities	Expenditures on innovation (without R&D)	Activities related to the acquisition or lease of property, plant and equipment	Software development and database activities	Marketing activities and building brand value	Activities related to the training of employees	Intellectual property activities	Engineering, design and other creative activities
		X_1_	X_2_	X_3_	X_4_	X_5_	X_6_	X_7_	X_8_	X_9_
1	Denmark	24871	19412	5459	1271	n/a	n/a	n/a	148	n/a
2	France	14954	10993	3961	379078	41914	27457	15611	2140	3216
3	Malta	5901	1543	4358	16083	7423	10,611	866	3460	1064
4	Spain	4343	2185	2158	11454	3028	3554	340	230	1347
5	Hungary	2551	1468	1082	7851	1940	1646	527	222	110
6	Poland	2247	1296	951	7881	1730	2301	593	255	138
7	Croatia	1815	682	1133	10832	1593	1861	1565	485	178
8	Slovakia	1588	754	833	4082	854	1317	201	73	300
9	Romania	1578	1476	102	7907	1463	996	63	0	504
10	Portugal	1376	953	423	4135	1307	1989	1087	152	242

Source: Own elaboration on the basis of Eurostat data [inn_cis11_exp], retrieved on May 4, 2021

*n/a*, data not available

Considering the expenditures on R&D, it should be noted that it dominated the remaining expenditures on innovation (excluding R&D). This was the case in 7 countries, with Romania having the greatest disproportion. Expenditures on R&D activities accounted for as much as 93.5% of total expenditures on innovation. Large disproportions also occurred in Denmark and France, which are leaders in this statistics. Such results prove the high activity of service enterprises in the field of R&D works carried out in these countries. It is a positive structure, as innovations resulting from the expenditures on R&D have a great chance of achieving a high level of innovativeness and thus building a strong competitive position of the service sector in these countries.

In the case of three of the surveyed countries, Slovakia, Croatia, and Malta, the proportions between R&D expenditures compared to non-R&D expenditures were reversed. This was most evident in the Maltese sector, where 73.9% of total expenditures on innovation activities was related to expenditures other than R&D.

When analyzing in detail the structure of expenses incurred on individual types of innovative activities (except R&D), it should be noted that the largest share was those related to the purchase or lease of tangible fixed assets (75.26%). Their highest value was recorded in the French service sector (379078 EUR/company), which definitely dominated over other countries. Countries such as Malta, Spain, and Croatia also showed high levels of outlays related to the purchase of fixed assets. In the service sector of these countries, the company spent more than 10000 EUR on the purchase or lease of property, plant, and equipment. In the case of services, tangible fixed assets are to a large extent building, structures or leased premises intended for the provision of services or the storage of goods. In addition, tangible fixed assets also include machinery and equipment used in the process of providing a given service. The lowest value of this type of expenditures was recorded in Denmark, and expenditures below 5 000 EUR/company was still identified in Portugal and Slovakia.

Expenditures on software development and database activities were the second most common type of innovation expenditures in the European service sector. However, their share among all expenditures on innovation is only 10.23% — almost seven times less than expenditures on tangible fixed assets. The shares of expenditures on other forms of innovative activity did not exceed 10%, and the lowest were in the case of engineering, design and other creative activities (1.19%).

When considering partial measures related to the effects of innovative activity, they should be divided into two sets. The first of them were indicators showing the level of innovativeness in the service sectors (X_10_– X_14_) of individual countries, presented in Table 3, while the second were indicators related to revenues obtained from product innovations (goods and/or services) by the countries of the service sector (will be discussed later in the study).

**Table 3:** The level of innovativeness in the service sector in the EU countries in 2018 (data in%)^a^

No	Country	Innovative enterprises	Product and/or business process innovative enterprises (including enterprises with abandoned/suspended innovation activities)	Business process innovation	Product innovation	Non innovative enterprises
		X_10_	X_11_	X_12_	X_13_	X_14_
1	Malta	2.80	2.67	2.49	1.67	4.74
2	Denmark	1.96	n/a	1.63	1.08	1.54
3	France	1.69	1.63	1.40	1.03	2.65
4	Croatia	1.64	1.62	1.44	1.15	1.47
5	Spain	0.87	0.81	0.70	0.36	3.25
6	Hungary	0.54	0.53	0.39	0.37	1.31
7	Romania	0.49	0.43	0.32	0.28	3.33
8	Portugal	0.47	0.46	0.41	0.34	0.78
9	Poland	0.45	0.42	0.37	0.21	1.69
10	Slovakia	0.37	0.35	0.28	0.16	1.02

Source: Own elaboration on the basis of Eurostat data: [inn_cis11_bas] and [inn_cis11_prod], retrieved on July 23, 2021

^a^data presented as a percentage of all service enterprises

*n/a*, data not available

The hierarchy of countries in Table 3 was made on the basis of the innovative enterprises index (X_10_) defining the percentage share of innovative enterprises among all service companies. Additionally, when analyzing the data presented in the table, it can be observed that the order of countries in the case of the X_11_ indicator related to innovative activity was the same as in X_10_. However, its values were slightly lower due to the fact that activities related directly to the production of goods and services were not included in this activity.

On the basis of the presented data, it should be noted that in most of the analyzed countries, non-innovative enterprises prevailed in the service sector. Such a situation occurred in eight of the surveyed countries. The worst ratio between the X_10_ and X_14_ indicators was identified in Romania, which is the weakest country in the entire EU also in terms of the total innovativeness indicator (Europejski Ranking Innowacyjności, 2018, 2018). The share of non-innovative companies amounted to 3.33% as much as 2.38 percentage point (p.p.) more than the innovative ones. A similarly unfavorable situation was also recorded in Spain — 3.25% non-innovative as compared to 0.87% innovators. Malta had the highest percentage of non-innovative service entities, where 4.74% of such companies in the sector were identified. Denmark was an exception to the discussed countries. It had a higher percentage of innovative companies (1.96%) as compared to entities that did not introduce innovations (1.54%). Croatia was the second country with a favorable relation between these indicators, but here the difference was slightly smaller and amounted to 0.17 p. p.

The X_12_ and X_13_ indicators allowed for a detailed analysis of the innovative activity of the service sector. Using them, the share of enterprises introducing product innovations and business process innovations in relation to service enterprises from the entire sector was presented. In the case of each country, business process innovations (mainly related to auxiliary functions) prevailed over product ones, which is a specific feature of the service sector, although as research shows, it does not always have to be this way (González-Blanco et al., 2018). When considering business process innovations, the best indicators were observed in Malta (2.49%) of all service companies implemented such as innovations, in Denmark (1.63%), and in Croatia (1.44%). Based on the data, it can be concluded that in 2018, a much greater percentage of enterprises introduced innovations in business processes than in the area of ​​product innovations, which, apart from products, also include services provided.

In the case of product innovations, the highest rates were again recorded in Malta (1.67%), Croatia (1.15%), and Denmark (1.08%). At the same time, it should be noted that innovative activity in the area of product innovations prevailed in the area of services, but not products having a material, tangible form.

Detailed analysis of business process innovations provided information on the introduced and improved innovative solutions (the list was not included in the text of the article due to the volume limitations of the work). The most common ones were those related to methods of improving and processing information and communication — 19.1% of all business process innovations. The second most popular changes were methods of organization or human resource management — 16.9%. The least popular innovations were those related to logistics — 9.96% of changes (Database [inn_cis11_spec], retrieved on July 26, 2021).

The second set of the analyzed indicators related to the effects were measures relating to revenues obtained from product innovations (X_15_–X_17_), which were differentiated according to the degree of their novelty on the market. These measures are presented per one service enterprise. The surveyed countries were ranked according to declining value of revenues obtained from new products on the market scale (Table 4).

**Table 4:** The level of revenues for new or significantly improved products due to the scale of new products (in euro/number of service company)

No	Country	New or significantly improved products that were new to the market	New or significantly improved products that were new to the firm	New or significantly improved products
		X_15_	X_16_	X_17_
1	France	27212	29802	57015
2	Denmark	26522	39468	65991
3	Spain	16368	35182	51550
4	Malta	12500	19327	31826
5	Romania	9006	9796	18803
6	Slovakia	7767	5198	12965
7	Hungary	6258	5470	11728
8	Croatia	4858	19371	24230
9	Portugal	4374	14199	18573
10	Poland	2650	4946	7596

Source: Own elaboration on the basis of Eurostat data [inn_cis11_prodt] retrieved on May 5, 2021

Revenue-related indicators allowed for the determination of financial benefits resulting from the introduction of product innovations. The highest value of new and improved products was identified in Denmark (65991 EUR/company). The next in the classification were France (57015) and Spain (51550). However, the service sectors of France and Denmark deserve distinction due to the high value of revenues obtained from new or improved products considered from the market perspective (27212 and 26522 EUR/company). The poorest financial results in the discussed scope, regardless of the scale of new innovations, were observed in Poland.

Generally, it can be observed that the smaller the scale and the level of novelty of the innovation, the greater the activity was identified in individual countries. The situation in Hungary and Slovakia was also worth noting, where service companies exceptionally obtained higher revenues from the sale of innovations new to the market than from novelties in the company itself, which could indicate a more expansive nature of innovation and this allows for a favorable assessment the level of innovative activity in this area.

Summarizing the part so far, which was based on the analysis of detailed indicators, it should be noted that the effects of innovative activity in the form of revenues and the level of innovativeness (expressed as a percentage of innovative enterprises) were at one of the highest levels among the same three countries. They include France, Denmark, Malta, as well as Spain in terms of the X_15_ indicator and Croatia of the X_10_ indicator.

On the basis of the conducted analyzes and comparing the data on the effects with the previously discussed level of expenditures, some analogies were noticed. In countries such as Malta, France, Denmark, and Spain, one of the highest indicators related to the overall expenditures on innovative activities X_1_ and measures X_2_ and X_3_ was recorded. At the same time, these countries dominated in terms of the effects obtained from innovative activities. It was noticed that the higher the level of expenditures on innovative activities (including R&D) was presented by the service sector of a given country, the higher the indicators related to the effects in the form of the level of innovativeness and from revenues from product innovations.

In this part of the analysis, it is worth mention that the researched countries varied in spite of the number of small and medium enterprises (SME). As an example in service sector in Malta, 1584 SME were identified, and on the other hand the number in Spain was 89660. It could be noted that in countries having more SME, the indicators characterizing innovative activity are ceteris paribus lower. Conducted analysis in range of the research as well as Spearman correlation rank has not confirmed such phenomenon. Results of correlation between volume of SME and indicator of innovative activities were statistical irrelevant.

### Identification and Classification of Internally Homogeneous Clusters in Terms of the Service Sector of the EU Countries Studied

**Fig. 1:**
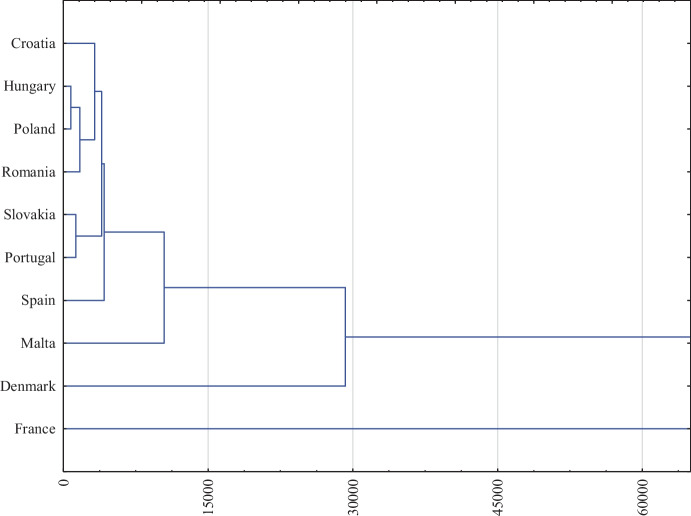
Typology of European Union countries according to the level of expenditures on innovative activities in the service sector in 2018. Source: Own elaboration on the basis of Statistica 13

The second stage of the research was to classify the service sectors of individual EU countries due to the similarity in terms of expenditures on innovative activities, as well as the effects resulting from them. Using the Statistica program, a cluster analysis was performed using the Ward’s method, as a result of which grouped research results were presented on a binary tree, which was visualized on a dendrogram. First, the analysis covered indicators related to expenditures on innovation — X_1_–X_9_ (Fig. 1). It should be added that the so-called cut-off line (it does not appear in the presented figure due to the adopted scale), i.e., the point with the greatest difference in distance between nodes, in the case of expenditures on innovation, was at the level of 350000 EUR/company.

Two large clusters of countries were identified on the basis of the binary tree. One group includes Denmark, Malta, and France, in which a relatively high level of outlays for innovative activities was identified as compared to other analyzed countries. Each of these countries was characterized by a specific and incomparable level of expenditures on innovation. This was also confirmed by the analysis of the Spearman’s rank correlation results. These countries were poorly (Denmark–Malta: 0.122) or highly correlated (France–Malta: 0.650), but the results were not statistically significant (Table 5).

**Table 5:**
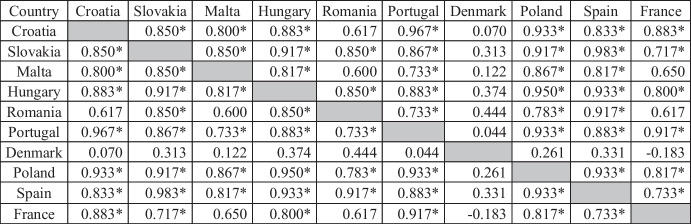
The value of the correlation of the Spearman order in terms of expenditures on innovative activities in the service sector of the EU countries in 2018

*Statistically significant correlation at the level of *p* = 0.95

Source: own elaboration on the basis of Statistica 13

Significantly smaller differences and closer clusters were observed in the case of other countries, which were characterized by lower spending. One cluster included countries such as Slovakia and Portugal, which on the basis of the binary tree, were found to be very similar in terms of inputs. This conclusion was also confirmed by the correlation at a very high level (0.867).

The second cluster is a group of closely related countries such as Hungary and Poland. An almost complete correlation of 0.950 was identified between them. The outlays in Poland were not so closely linked with any other country as with Hungary. On the basis of the dendrogram, it can also be concluded that outlays on innovative activities in Poland and Hungary were definitely less related to those in Romania or Croatia. This observation was also confirmed by the correlation analysis, in which Poland’s relationship with Croatia at the level of 0.933 or with Romania at 0.783 was noted. In the case of Hungary and Croatia, the correlation was at the level of 0.883 and in the case of Hungary and Romania was 0.850.

Based on the comparison of Spearman’s rank correlation results (Table 5), it can be seen that only the value of outlays in Denmark was not statistically related to other countries. In general, it can be concluded that the clusters of countries closest to each other in terms of expenditures observed in the binary tree were also identified and confirmed by an objective statistical method using the Spearman’s rank correlation index.

**Fig. 2:**
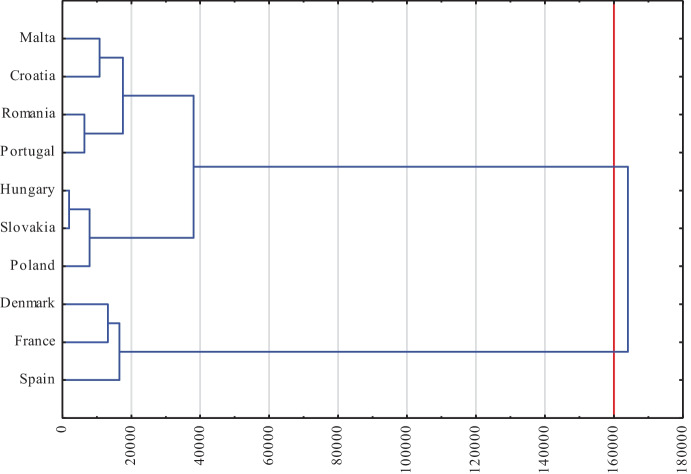
Typology of European Union countries according to the results of innovative activities in the service sector in 2018. Source: own elaboration on the basis of Statistica 13

The analysis with the use of the dendrogram and the Spearman’s rank correlation index was carried out in relation to the effects achieved by the EU service sector as part of innovative activities. In this case, the cut-off line was set at 160000 (Fig. 2).

There are two large clusters in the binary tree chart. One should include Spain, France and Denmark — forming a smaller cluster — i.e., countries with the best indicators of the effects of innovative activities. The effects of the conducted innovative activity in the case of the last two of the above-mentioned countries were statistically significantly correlated with each other, which were confirmed by the performed statistical analysis (Table 6). There was an almost full correlation at the level of 0.964. The remaining seven countries created a second cluster, in which two more smaller subgroups were identified. The first of them were Hungary and Slovakia, and Poland joined them. The level of the effects of innovative activity in all of these three countries was statistically significantly related to each other. The Spearman coefficient confirmed almost full correlation between: Hungary–Slovakia (0.976), Hungary–Poland (0.952), and Poland–Slovakia (0.976). Based on the dendrogram, four countries were classified into the second group and created two smaller clusters. The first one includes Romania and Portugal, while the second one includes Malta and Croatia (almost full, statistically significant correlation at the level of 0.929).

**Table 6:**
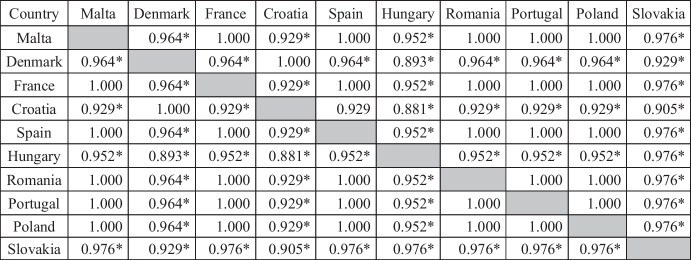
The value of the correlation of the Spearman order in terms of the effects of innovative activities in the service sector of EU countries in 2018

*Statistically significant correlation at the level of *p* = 0.95

Source: own elaboration on the basis of Statistica 13

## Estimation of the Level of Expenditure on Innovative Activities and the Effects Achieved as a Result

**Fig. 3:**
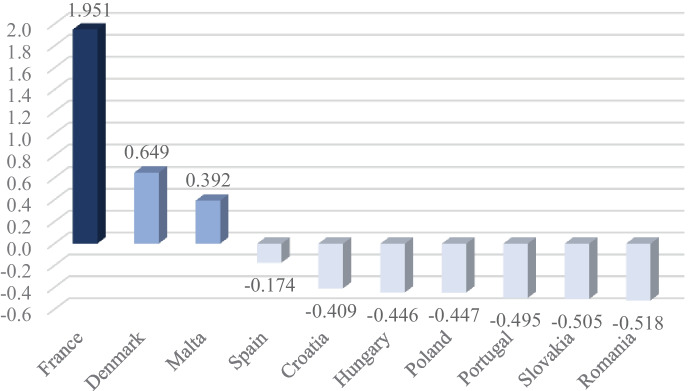
Perkal’s index — level of expenditures on innovative activities in the EU service sector. Source*:* own elaboration on the basis of of MS Excel and Eurostat data [inn_cis11_exp], retrieved on May 4, 2021

The third stage of the conducted research was the assessment of the degree of use of inputs in comparison to the effects achieved by the service sector of individual countries. Perkal’s index was used for this. Considering the results of research on expenditures and using the grouping criteria presented in the methodological part of the work (Table 1), it can be concluded that in general the highest level of expenditures on innovative activities was recorded in France: PI = 1.951 (Fig. 3). This situation was certainly influenced by the value of expenditures on activities related to the purchase of tangible fixed assets incurred by the French service sector, as well as a significant disproportion compared to other countries. Innovation expenditures at an average level were observed in Denmark (PI = 0.649) and in Malta (PI = 0.392). The other seven countries had a low Perkal’s index.

**Fig. 4:**
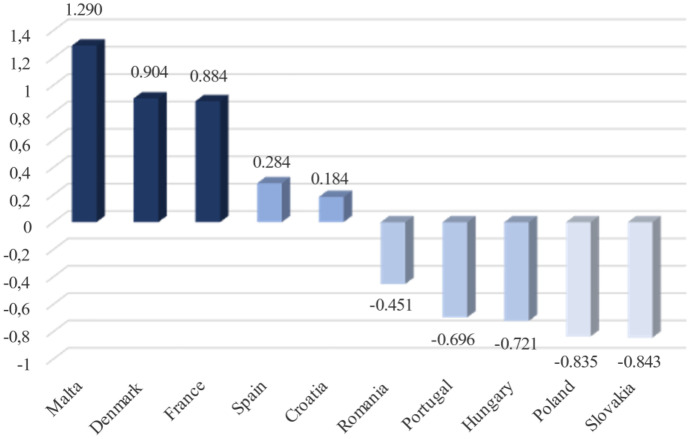
Perkal’s index — the level of effects resulting from the innovative activity conducted in the EU service sector. Source: own elaboration on the basis of MS Excel and Eurostat data [inn_cis11_prodt] retrieved on May 5, 2021 and [inn_cis11_bas], retrieved on July 8, 2021

The value of the Perkal’s index in the case of effects was more diversified than it was in relation to expenditures on innovative activities. Four levels are distinguished. The best indicators determining the effects of innovative activity were characteristic of the Maltese service sector: PI = 1.290 (Fig. 4). Other countries where the PI level was also defined as high were Denmark (PI = 0.904) and France (PI = 0.884). In the next two countries, i.e., Spain and Croatia, the average level of the measure in question was recorded. The remaining countries were classified as low or very low in terms of PI. A characteristic feature of the index in these countries was its negative values, the lowest being identified in Poland (PI = − 0.835) and Slovakia (PI = − 0.843). These results showed that in general, the worst effects of innovative activity were recorded in these countries.

By comparing and summarizing the values of the Perkal’s index, it was possible to determine the degree of use of expenditures on innovation in the service sectors of individual countries. It can be concluded that the outlays were most effectively used by service enterprises in countries such as Malta and Denmark, where the average level of outlays translated into a high level of innovativeness achieved as a result of them (Table 7).

**Table 7:** The level of effects in relation to the expenditures used for innovative activities in the service sector of the EU countries in 2018

Country	Perkal’s index level for inputs	Perkal’s index level for effects	The relation of effects to inputs
Malta	Medium	High	Positive — good results in relation to the expenditures incurred
Denmark	Medium	High	
Spain	Low	Medium	
Croatia	Low	Medium	
France	High	High	Neutral — effects adequate to the expenditures incurred
Romania	Low	Low	
Portugal	Low	Low	
Hungary	Low	Low	
Slovakia	Low	Very low	Negative — low/weak effects in relation to the expenditures incurred
Poland	Low	Very low	

Source: own study based on research

The second group with a favorable relationship between the expenses and the benefits derived from them was Croatia and Spain, where the outlays were identified at a low level and the effects at an average level. The worst situation in terms of the degree of use of inputs in relation to the effects was observed in Poland and Slovakia, where a low level of expenditures on innovation was recorded and which, additionally, was not used effectively. The effects achieved by the service sectors of these countries were classified as very low. In the case of other countries (France, Romania, Portugal, Hungary), it can be concluded that the outlays on innovative activities were used adequately to the effects achieved as a result of them.

## Conclusions

The numerous studies conducted so far in the scope of the described issues separately treat the innovative potential understood, for example, as expenditure on innovative activity and the effects obtained as a result of using this potential (conducting innovative activity using the possessed potential). There are few studies taking into account the two issues mentioned simultaneously, i.e., identifying the efficiency of using inputs in innovative activities. It is especially not a popular research area in the case of the service sector of enterprises. This is a real research gap, which to some extent — though certainly insufficient — fills this study. In relation to the above, the results of the conducted analyzes were primarily a source of information and knowledge on the effectiveness of the use of expenditure allocated to various types of innovative activities, conducted by service entities of individual European Union countries.

In connection with the research gap identified above, the purpose of the research and analyzes presented in this study was to assess the level of innovative activity in the service sector in the EU Member States, in terms of incurred expenditure and achieved results. The indicators relating to both categories were analyzed, and the verification and evaluation were carried out in a three-stage verification process with a complex degree of detail. On the basis of the conducted research and thanks to the application of an extensive research approach, it was possible to draw five conclusions.

Considering the expenditures on innovative activities, on the basis of the analysis of detailed indicators, it can first be concluded that the highest level was characteristic of Denmark, especially in terms of total expenditures and expenditures on R&D. In other countries, much lower outlays were recorded. Considering the expenditures on innovative activities in terms of their allocation (types of innovative activity), the French service sector was one of the highest expenditures. In general, the service sectors of countries such as Portugal, Romania, and Slovakia were the worst in terms of expenditures.

The presented conclusions have been extended to the binary tree analysis. On its basis, a second conclusion from the research can be drawn that the service sectors of the surveyed countries were classified into two groups. One includes countries that are highly diversified in terms of expenditures, such as Denmark, France, and Malta. On the other hand, the other countries with smaller subgroups with greater similarity were classified into the second group. This was confirmed by the Spearman’s rank correlation coefficient, on the basis of which statistically significant, almost complete, and very high correlations were observed. On the basis of a comprehensive approach to the issue of expenditures on innovative activities, it was possible to draw a third conclusion that countries with a high level of investment were at the same time leaders in the innovation ranking, such as Denmark and countries catching up with leaders, such as France. The countries of *moderate* and *weak innovators* were characterized by low expenditures on innovation (including Spain, Portugal, or Poland). The presented approach to the issue of expenditures on innovative activities made it possible to achieve the first specific objective set out in the research.

The second assessed “dimension” of innovative activity was the output elements in the form of effects. Based on the analysis of detailed indicators, it was possible to make a fourth conclusion that the best results of innovative activities in 2018 were achieved by the service sectors in Denmark and France, as well as in Malta. The first two countries were characterized by the highest level of expenditures on innovation; moreover, in the innovation ranking, they were included in the group of leaders and countries advancing the leaders, respectively. At the same time, these countries were more closely correlated with each other in terms of effects than in the case of spending. The worst situation was recorded in Slovakia and Poland.

Cluster analysis for effects identified three clusters of countries. One of them included Denmark, France, and Spain. The second set was Malta, Croatia, Romania, and Portugal and the third Hungary, Slovakia, and Poland. Belonging to these clusters was confirmed by the Spearman’s rank correlation coefficient, the values of which in the vast majority of cases were statistically significant. The interpretation conducted based on a binary tree confirmed the results of previous index analyzes, and the Spearman’s rank correlation objectively proved the correctness of aggregating countries into individual clusters. Comprehensive analysis and evaluation of the effects of the conducted innovative activity are allowed for the achievement of the second detailed objective.

On the basis of the Perkal’s index, it can be concluded (fifth conclusion) that the level of expenditures on innovative activities did not always correspond to the effects achieved as a result. When assessing the effectiveness of innovative activity in the service sector of individual countries, they can be conventionally divided into three groups. In the first one there was a positive relation of the effects to the incurred expenditures on innovation. This group includes countries such as Malta, Denmark, Spain, and Croatia. The level of the effects of innovative activity in these countries was relatively higher in relation to the amount of expenditures. The second group consisted of countries such as France, Romania, Portugal, and Hungary, where the use of outlays was adequate in relation to the effects achieved on their basis. The last group, in which the effectiveness of innovative activity was described as unfavorable (negative), was made up of only two countries: Slovakia and Poland. The comparison of the indicators related to expenditures on innovation with the effects made it possible to achieve the third specific objective on the relationship between inputs and effects identified in individual EU countries.

The presented research may be implicit in economic practice. Firstly, they constitute a collection of information for service enterprises on the level of innovation of the service sector in the country in which they operate. The described research can be treated as some “average” results of the level of innovative activity of the service sector of a given country. Entrepreneurs can use the research results to verify their innovative position by comparing the level of innovative activity of the entire sector with the innovativeness of their company. Secondly, this work, together with the results of the analyses carried out, can be an impulse for the creation of many studies and represents a significant potential for the future in the field of innovation. The comparison of the level of use of inputs in relation to the effects achieved by individual countries, based on the Perkal’s index, provided many interesting inspirations for creating ideas for the implementation of research in the future. The conducted analysis in this area may also constitute a source of new and original knowledge, resulting from the little popularized approach to the issue of the effectiveness of innovative activities. An extremely interesting research area may be the identification of determinants of the situation in the service sectors of countries that most effectively used expenditure on innovative activities (Malta, Denmark, Spain, or Croatia). Examples of service sectors in these countries can provide a source of good practice in terms of the effective use of funds allocated to innovative activities. As a consequence, another area that is extremely interesting from the cognitive point of view is the identification of conditions — it seems that mainly barriers — which make the level of the achieved results from innovative activities inadequate (disproportionate) in some of the surveyed countries (Slovakia, Poland) to the expenditure allocated to their acquisition. In the case of both of the above-mentioned research areas (defining barriers and determinants of using outlays for innovative activities), it should be mentioned that the identified situation could have been influenced by the potentially diversified innovation policy applied in the countries mentioned, including in particular the forms, availability of support instruments, and the experience of individual countries in terms of its implementation. The impact of the policy on the effectiveness of the inputs used could be the subject of a separate study.

The last extremely inspiring area of research may be the analysis of the level of innovation activity, the period of the coronavirus pandemic, and the observation of possible differences. It will then be possible to observe potential changes in the level of innovation in the service sector, which seems to have suffered significantly from the pandemic. Such a state was observed, for instance, in Poland and presented on the case of the dynamics of changes in the basic indicators of the development of the sector (Dominiak, 2022, 130-131). On the other hand, the impact of the epidemic situation should not be generalized to the entire service sector. Not all industries suffered to the same degree, for example section J, K or even G compared to sector I, which was most affected by the pandemic (Dominiak, 2022, 130-134). The business services sector in Poland has even progressed, and the pandemic has accelerated the development of the business services sector (ABSL, 2022).
